# Energy Transfer in Eu_*x*_Gd_1–*x*_(OH)_3,am_

**DOI:** 10.1021/acsomega.6c02791

**Published:** 2026-07-07

**Authors:** Sophie Zenker, Nicolas Jahn, Michael U. Kumke

**Affiliations:** University of Potsdam, Institute of Chemistry (Optical Sensing and Spectroscopy), Karl-Liebknecht-Str. 24-25, 14476 Potsdam, Germany

## Abstract

This study examined the luminescence properties and quenching mechanism in amorphous Eu­(OH)_3_ and Eu_*x*_Gd_1–*x*_(OH)_3_ under alkaline conditions (pH 12.5) representative of cement porewaters. Different Gd­(III) contents were used to alter the average Eu­(III)–Eu­(III) distances in the amorphous solids. Gd­(III) was considered “inert” due to the similar ionic radius and lack of spectral overlap between Eu­(III) and Gd­(III). The Eu­(III) luminescence was investigated using time-resolved laser-induced luminescence spectroscopy (TRLFS), and the luminescence decay curves were analyzed according to the Inokuti–Hirayama model. It was found that the luminescence quenching in the investigated materials is primarily caused by energy migration between Eu­(III) ions best described by dipole–dipole interactions with an estimated critical Eu­(III)–Eu­(III) distance *R*_0_ of 5.8 ± 0.3 Å. Temperature-dependent experiments revealed that the quenching efficiency significantly decreased below 250 K, indicating that Eu­(III) ions in thermally populated ^7^F_1_ and ^7^F_2_ levels take part in the energy migration. Employing the RET pair Tb­(III)/Eu­(III) in Tb_*x*_Eu_0.0005*x*_Gd_1–*x*_(OH)_3_ offered additional evidence that interlanthanide energy transfer occurs in the amorphous lanthanide hydroxides. In addition, concentration-independent quenching processes, such as energy transfer to vibrational overtones of OH^–^ or H_2_O, were observed. These findings contribute to understanding the photophysics of amorphous lanthanide hydroxides and may also offer insights into other Eu­(III)-rich phases that may form under conditions relevant for nuclear waste repositories.

## Graphical abstract

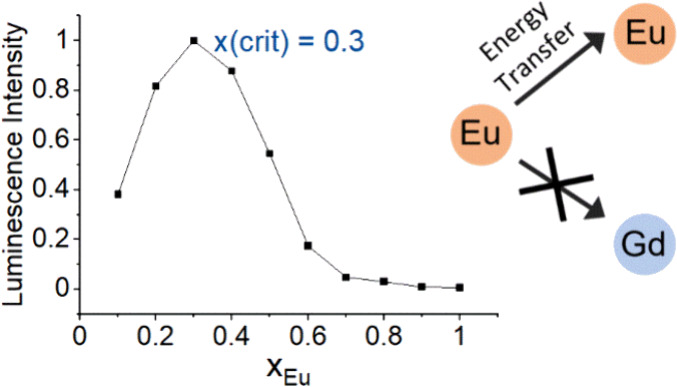

## Introduction

Europium (Eu­(III)) is successfully used as a luminescence probe in environmental and life science applications for decades.^1–4^ Its outstanding luminescence properties, such as the relatively narrow, well-separated luminescence bands and the nondegenerate ground-state ^7^F_0_ and luminescence level ^5^D_0_, render it useful e.g. as a luminescence donor in sandwich fluorescence immunoassays, as a substituent for Ca­(II) in protein research and as a nonradiotoxic analog for trivalent actinides in nuclear waste research.^5–9^ It is widely accepted to use Eu­(III) as natural analog for Am­(III) and Cm­(III) because of their very comparable chemistry without the challenge of radioactivity.

Since Am­(III) and Cm­(III) appear as part of nuclear waste, understanding their behavior under conditions present in a nuclear waste repository is vital for the safety case. In many layouts of repositories for nuclear waste cement is used either as part of a technical barrier to hinder the migration of radionuclides or as part of the building structure. The pore water in equilibrium with the cement is alkaline, with the exact pH and composition of the pore water depending both on the cement composition and on the stage of cement degradation due to pore water exchange processes. Fresh cement has a high pH of >13 and the pore water is dominated by highly soluble alkali hydroxides, while in a second degradation stage (stage II) the pH of the pore water is determined by the solubility of portlandite and buffered around pH = 12.5. In the course of further leaching (especially of Ca^2+^ ions) the degradation of the cement progresses and subsequently the pH of the pore water decreases to ∼9 at the end of the third degradation stage.^10^

For trivalent lanthanides (Ln­(III)) and actinides (An­(III)) in contact with cement pore water the formation of hydroxo complexes is inevitable. In Figure 1 a speciation diagram for Eu­(III) in water calculated from the ThermoChimie v12a Davies database^11^ is shown. For pH > 6 Eu­(III) ions undergo hydrolysis of H_2_O ligands yielding mononuclear and multinuclear hydroxides, some of which have very low solubility.^11,12^ For a cement system at degradation stage II with a pH of ∼12.5 the formation of amorphous Europium­(III) hydroxide (Eu­(OH)_3,am_) is expected to occur upon contact with the pore water. Consequently, for a sound speciation based on TRLFS (time-resolved laser-induced luminescence spectroscopy) data on the photophysics of hydroxo species is of importance.

**Figure 1:**
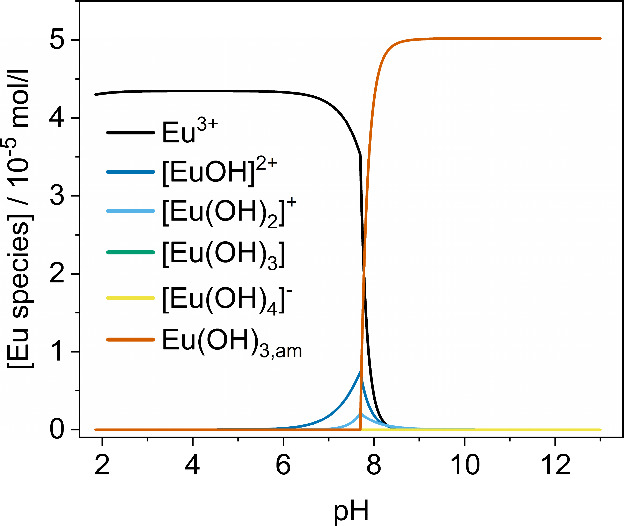
Calculated speciation of Eu­(III) in equilibrium with Eu­(OH)_3,am_ as a function of the pH using the ThermoChimie v12a Davies database^11^ in PHREEQC^13^ ([Eu­(III)]_tot_ = 5 × 10^–5^ M, *T* = 25 °C, pH was adjusted using NaOH and HCl).

In the literature TRLFS data on the different Eu­(III) hydroxo species can be found.^14–18^ However, for high pH values and Eu­(III) concentrations, at which Eu­(OH)_3,am_ dominates the speciation (see Figure 1), the data are sparse and inconclusive. A major challenge is that one has to deal with dispersions containing colloidal Eu­(III) species. TRLFS measurements of dispersions require strict control of measurements conditions, e.g., the use of a stirrer to get the particles into the light path and to avoid sedimentation of the particles during the measurement. Furthermore, due to the amorphous character of Eu­(OH)_3,am_, its luminescence properties (e.g., the luminescence spectrum and luminescence decay time) appear to be also dependent on the sample conditions, such as pH, temperature and aging time. As an example, for precipitated Eu­(III) hydroxide luminescence decay times of 21.6 ± 3.3 μs,^16^ 46 ± 2 μs,^19^ 90 ± 5 μs^20^ and 158 ± 7 μs^15^ have been reported. Takahashi et al. observed luminescence decay times between 10 and 110 μs for Eu­(OH)_3,am_ at different pH values.^17^

Some of the reported luminescence decay times are distinctly shorter than the one of the Europium­(III) aquo ion (Eu­(H_2_O)_8–9_). A common explanation for the relative short luminescence decay times in aqueous media is, that the Eu­(III) luminescence is quenched by an energy transfer to a vibrational overtone of the O–H vibrations of coordinated water molecules or hydroxide ions. An empirical relationship between the number of water molecules in the first coordination sphere of Eu­(III) and the luminescence decay time has been developed by Horrocks et al. and adapted by various other researchers.^21–23^ Since in Eu­(OH)_3,am_ there are fewer O–H vibrators in the coordination environment of each Eu­(III) ion than in the Eu­(H_2_O)_8–9_ ion, the short luminescence decay times reported in literature cannot be explained solely by the quenching from O–H vibrators. Instead, in Eu­(OH)_3,am_ a close proximity of Eu­(III) ions is expected giving rise to the possibility of concentration quenching via Eu­(III) → Eu­(III) energy transfer processes. A common phenomenon in lanthanide-rich materials is cross-relaxation.^24–26^ Here, an excited lanthanide ion transfers a part (but not all) of the excitation energy to another ion of the same element, which depopulates the initially excited level, and thus quenches luminescence from this level. However, this process is only possible, if suitable energy levels with matching energy differences are available. For Eu­(III), the energy difference between the ^5^D_0_ and the ^7^F_6_ level (∼12000 cm^–1^) is significantly larger than the energy difference between the ^7^F_0_ and ^7^F_6_ level (∼5000 cm^–1^), hence no cross-relaxation between an ion in the ^5^D_0_ level and one ion in the ^7^F_0_ level can occur.^4,24,27^ Nevertheless, cross-relaxation involving three Eu­(III) ions in the form of ^5^D_0_ + 2 ^7^F_0_ → 3 ^7^F_*J*_ (*J* = 4–6, with the assistance of phonons to overcome the remaining energy mismatch) has been proposed by Van Uitert and Johnson, and Yusenko et al., although the probability of such a transfer is expected to be low due to the involvement of three ions and the energy mismatch of ∼2000 cm^–1^.^24,27^ Furthermore, cross-relaxation between two excited Eu­(III) ions in the ^5^D_*J*_ manifold could occur, which would result in one Eu­(III) ion in a higher excited state (which could still exhibit luminescence after relaxation to ^5^D_0_) and one Eu­(III) ion in a ^7^F_*J*_ level, from which no luminescence occurs.^26,28,29^ Due to the low extinction coefficient of Eu­(III) however, the quota of excited Eu­(III) ions is generally expected to be small, meaning that only rarely two excited Eu­(III) ions will be next to each other. Another quenching pathway is that the excitation energy could migrate from one ion to the next until it is irreversibly transferred to a quenching center, such as impurity ions or defect sites.^30–37^ The higher the Eu­(III) content of the material, the more Eu­(III) ions can be connected to quenching centers through a series of Eu­(III) → Eu­(III) energy transfer steps, which might occur via (super)­exchange^30,31,34,35^ or via multipolar interaction.^32,33,36^

While the concentration quenching of Eu­(III) has been studied in a variety of crystalline materials,^25,31–33,38^ to this date not much has been reported on amorphous Eu­(III) compounds, such as Eu­(OH)_3,am_, which appear in environmental studies such as the sorption of Eu­(III) in cement systems. The present study aimed to close this gap by investigating the luminescence of Eu­(OH)_3,am_ and of Eu_*x*_Gd_1–*x*_(OH)_3,am_ at pH 12.5 which is typical for cement systems at alteration stage two. Gd­(III) was used as a spectroscopically inactive ion to achieve different Eu­(III) contents in the solids (equal to a variation in the average distance between Eu­(III) ions in the solid), as Gd­(III) has a similar ionic radius and the same charge as Eu­(III), but no energy transfer from Eu­(III) to Gd­(III) is possible. Furthermore, a mixed hydroxide of Tb­(III)/Eu­(III) was investigated to further test the probability of interlanthanide energy transfer processes within the amorphous lanthanide hydroxides and the potential pathways for the deactivation of the Eu­(III) luminescence are discussed. For selected samples also the temperature dependence of the luminescence was investigated in order to further evaluate the mechanism behind the observed luminescence quenching.

## Methods

EuCl_3_, TbCl_3_ (both anhydrous, 99.99% trace metal basis, Sigma-Aldrich, USA), GdCl_3_·6H_2_O (99.999% trace metal basis, Sigma-Aldrich, USA) and NaOH (≥98%, Sigma-Aldrich, USA) were used without further purification. To avoid the formation of carbonates, all samples were prepared in a glovebox under N_2_ atmosphere using deionized water (18.2 MΩcm; Millipore GmbH, Germany), which had been purged with Ar overnight prior to use.

### Sample Preparation

Stock solutions were prepared by dissolving the desired amount of the lanthanide­(III) chloride in hydrochloric acid at pH 5. To prepare the Eu_*x*_Gd_1–*x*_(OH)_3_ samples, stock solutions of Gd­(III) and Eu­(III) were mixed at the desired molar ratio (0.1 ≤ *x* ≤ 1.0) at pH 5. Afterward, an appropriate amount of the lanthanide mixtures was transferred to PMMA cuvettes (Sarstedt AG & Co. KG, Germany) and NaOH solution was added to yield a solution pH value of 12.5 ± 0.1 (pH electrode and pH meter: Orion Star, Thermo Fisher Scientific Inc., USA) and a total lanthanide concentration (of solid and supernatant combined) of 5 × 10^–5^ M. For T-dependent measurements of Eu­(OH)_3_, a total concentration of 1 × 10^–3^ M was used due to signal constraints in these kinds of experiments. In all samples, the precipitation was nearly quantitative and the lanthanide concentration in the supernatant was below the LOD of the TRLFS setup (1 × 10^–7^ M). The lanthanides were mixed at low pH beforehand to favor the formation of mixed hydroxides over pure Eu­(III) and Gd­(III) hydroxide particles. Before the TRLFS measurements, the samples were left at pH = 12.5 ± 0.1 to equilibrate for 3 weeks, during which the analytes precipitated as colorless solids. To investigate a potential Tb­(III) → Eu­(III) energy transfer, additionally Tb_*x*_Eu_0.0005*x*_Gd_1–*x*_(OH)_3_ samples were prepared in an identical manner with a Tb­(III) stock solution that contained a small amount of Eu­(III) (Eu/Tb molar ratio ∼0.05 mol %).

### Luminescence Spectroscopy

Time-resolved laser-induced luminescence spectroscopy (TRLFS) was carried out with the dispersions, using PTFE micro stirring rods (Rotilabo, Carl Roth GmbH + Co. KG, Germany) together with a stirring unit (CuV–O–Stir 333, HELLMA GmbH & Co. KG, Germany) to whirl up the solid particles into the light path.

Two different TRLFS setups were used depending on the sample series. Measurements for Eu­(III) at room temperature were performed using a pulsed 20 Hz Nd:YAG laser (Quanta Ray, Spectra Physics, USA) in combination with an OPO (GWU-Lasertechnik, Germany) set to 394 nm (corresponding to the ^5^L_6_ → ^7^F_0_ transition of Eu­(III)). The detection system consisted of a spectrograph (Shamrock 303i, Andor Technology, Oxford Instruments, United Kingdom) with a 300 lines/mm grating (blaze wavelength: 500 nm) and an ICCD camera (iStar DH734-18H-13, Andor Technology, Oxford Instruments, United Kingdom).

To explore the temperature dependence of the quenching process, a sample of Eu­(OH)_3,am_ was tempered at 4 K, 50 K, 210 K, 250 and 293 K during TRLFS measurements using a cryostat system consisting of a helium compressor (Sumitomo Heavy Industries Ltd., Germany) and a 331 temperature controller (Lake Shore, USA) as well as a turbo-vacuum pump (Leybold Vacuum Turbolab 80, Oerlikon, Germany) to evacuate the sample chamber. Due to the poor detection limit of the temperature-dependent setup, a higher Eu­(III) concentration of 1 × 10^–3^ M and high number of accumulations were needed for this experiment. Hence, to limit the amount of materials and measurement time, only the molar ratio with the highest concentration quenching (*x* = 1) was investigated at different temperatures.

The temperature-dependent measurements, as well as the TRLFS measurements of the Tb_*x*_Eu_0.0005*x*_Gd_1–*x*_(OH)_3_ samples, were performed using a pulsed 10 Hz Nd:YAG laser (Quanta Ray, Spectra Physics, USA) and an OPO (GWU-Lasertechnik, Germany) for excitation together with a Kymera 328i spectrograph (Andor Technology, Oxford Instruments, United Kingdom) with a 300 lines/mm grating (blaze wavelength: 760 nm) and an ICCD camera (iStar sCMOS DH320T-18F-93, Andor Technology, Oxford Instruments, United Kingdom) for detection. The Tb_*x*_Eu_0.0005*x*_Gd_1–*x*_(OH)_3_ samples were excited at 377 nm to primarily excite the Tb­(III) ions and the luminescence of both Tb­(III) and Eu­(III) was recorded.

With both setups the luminescence spectra were recorded using the box-car technique with the measurement parameters summarized in Table 1. For Eu­(III) additionally kinetic series were measured at all investigated temperatures using a linear increasing gate step, that was individually chosen for every sample. After the measurements, the spectra were corrected for the spectral sensitivity of the ICCD camera and the efficiency of the grating and a background correction was performed.

**Table 1:** Measurement Parameters for the Different TRLFS Experiments

	Eu_*x*_Gd_1–*x*_(OH)_3_	Eu_*x*_Gd_1–x_(OH)_3_	Tb_*x*_Eu_0.0005*x*_Gd_1–*x*_(OH)_3_
	293 K	4–293 K	293 K
excitation wavelength [nm]	394	394	377
initial delay [μs]	10	10	10
gate width [μs]	1000	1000	1000
input slit width [μm]	200	25 or 400	400
number of accumulations	100	50	500

### Characterization with EDX

After the TRLFS experiments, the Eu_*x*_Gd_1–*x*_(OH)_3_ samples were used for energy dispersive X-ray spectroscopy (EDX) analysis. The precipitate was transferred onto a carbon film and dried over silica gel under N_2_ atmosphere. After drying, the samples were coated with carbon and EDX analysis was carried out with a JEOL JSM-6510 SEM (JEOL, Germany) equipped with a W cathode at 15 kV and an INCAx-act EDX detector (Oxford Instruments, UK).

## Results and Discussion

### Characterization with EDX

In order to check the molar ratio of the two Ln­(III) ions as well as the amount of impurity ions, that might influence the luminescence properties, the Eu_*x*_Gd_1–*x*_(OH)_3_ samples were investigated by EDX. For each sample, at least 15 different spots were analyzed. The averaged Eu­(III) content *x*_Eu_ = [Eu]/([Eu] + [Gd]) is shown in Table 2. It is apparent, that the experimentally determined Eu­(III) content by EDX is in very good agreement with the one expected based on the Eu­(III) to Gd­(III) ratio in the precursor solution. Furthermore, a nearly identical value for *x*_Eu_ was determined at each measured spot in the samples.

**Table 2:** Composition of the Eu_*x*_Gd_1–*x*_(OH)_3_ Samples^a^

Nr.	[Eu]_0_/1 × 10^–5^ M	[Gd]_0_/1 × 10^–5^ M	x_Eu_(theo.)	x_Eu_(EDX)
1	50	0	1.0	1.00 (contains no Gd)
2	45	5	0.9	0.90 ± 0.02
3	40	10	0.8	0.79 ± 0.01
4	35	15	0.7	0.68 ± 0.02
5	30	20	0.6	0.59 ± 0.02
6	25	25	0.5	0.50 ± 0.02
7	20	30	0.4	0.40 ± 0.01
8	15	35	0.3	0.29 ± 0.01
9	10	40	0.2	0.20 ± 0.01
10	5	45	0.1	0.11

a [Eu]_0_ and [Gd]_0_ denote the initial Eu­(III) and Gd­(III) concentration in the precursor solution before precipitation, respectively. *x*_Eu_(theo.) is the expected molar fraction of Eu­(III) in the precipitate based on the Eu­(III) and Gd­(III) ratio in the precursor solutions, while *x*_Eu_(EDX) denotes the molar fraction determined with EDX (averaged over approximately 15 measurement points on each sample).

Na, Cl, Si, Al and Ge were found as impurities. However, it is unclear, if the impurity ions were embedded in the Eu_*x*_Gd_1–*x*_(OH)_3_ particles during the TRLFS measurements or if they precipitated from the remaining supernatant in the drying process after the TRLFS experiments were already completed. None of the observed impurities will quench the Eu­(III) luminescence. Other lanthanides or d-metal ions, which could potentially act as quenching centers, were not detected.

### Luminescence Spectra of Eu_*x*_Gd_1–*x*_(OH)_3_ at 293 K

Figure 2 displays the raw (A) and to the area of the ^5^D_0_ → ^7^F_1_ peak normalized (B) luminescence spectra of the mixed Eu_*x*_Gd_1–*x*_(OH)_3_ samples taken from time-resolved emission measurements with 0.1 ≤ *x* ≤ 1 at a gate delay of 10 μs at *T* = 293 K. No change in the spectral features of the Eu­(III) luminescence spectra was observed upon changing the Eu­(III) molar ratio (see Figure 2B), indicating that the chemical environment at the location of the Eu­(III) ions is identical in all Eu_*x*_Gd_1–*x*_(OH)_3_ samples. However, the observed luminescence intensity varied significantly with the Eu­(III) content, as can be seen in Figure 2A. For *x* ≤ 0.3 the luminescence intensity increases with increasing *x* (as expected in the absence of luminescence quenching). At *x* = 0.3 the luminescence intensity reaches its maximum and decreases at higher Eu­(III) content. We attributed this effect to the presence of concentration quenching due to Eu­(III)–Eu­(III) energy transfer, which becomes effective at higher Eu­(III) contents. However, in case of dispersions comparing the absolute luminescence intensities is tricky due to scattering and other effects influencing the observed luminescence intensity. A major factor for errors here is a fluctuating concentration (and maybe size) of Eu_*x*_Gd_1–*x*_(OH)_3_ particles in the observation volume during the luminescence measurements. Although we tried to minimize these effects by using astrict protocol for sample preparation (vide supra) and stirring the samples during measurements, the alterations in the luminescence intensities can only be seen as a first indicator for the concentration quenching (vide infra).

**Figure 2:**
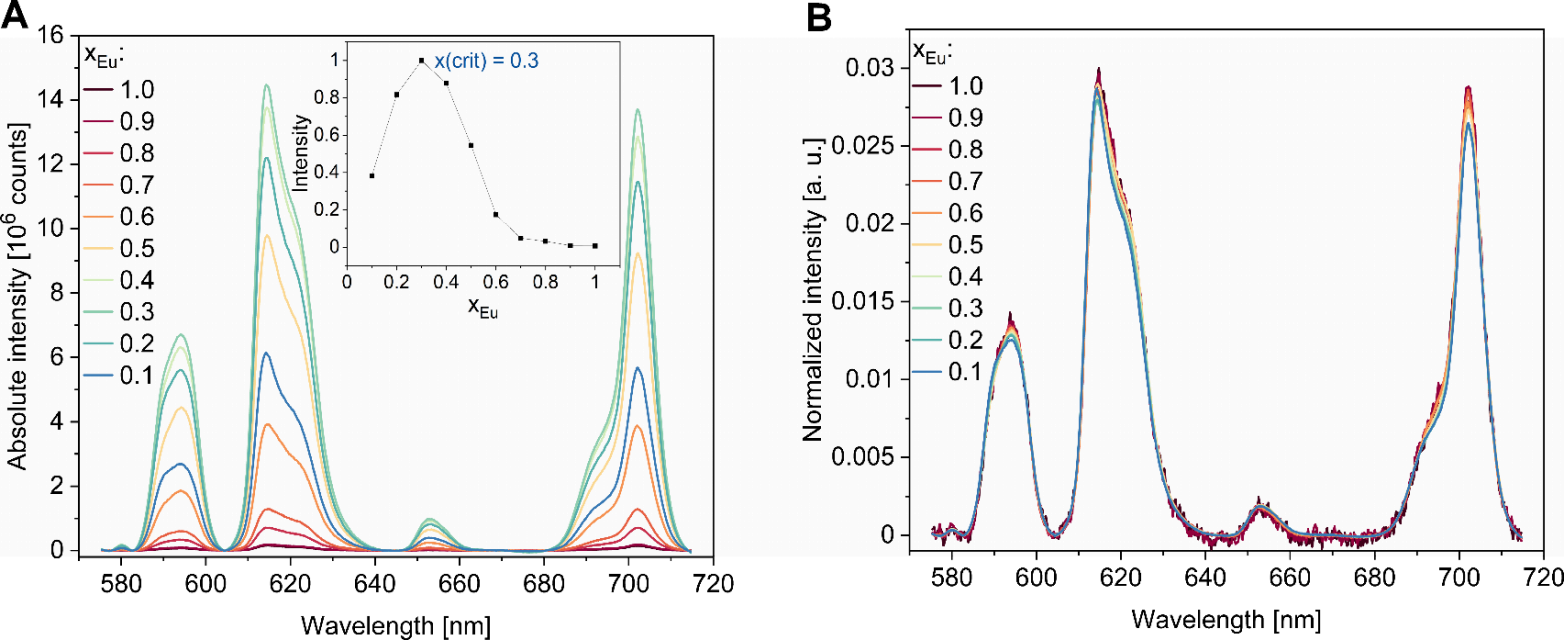
Luminescence spectra of Eu_*x*_Gd_1–*x*_(OH)_3_ at 293 K at a gate delay of 10 μs with different Eu­(III) contents. The spectra are shown with absolute luminescence intensities (A) and normalized to the area of the ^5^D_0_ → ^7^F_1_ transition (B). The inset in panel (A) shows the luminescence intensity (integrated over the whole spectrum) at each molar ratio of Eu­(III). The maximum luminescence intensity is reached at *x*_crit_ = 0.3.

### Tb­(III) → Eu­(III) Energy Transfer in Tb_*x*_Eu_0.0005*x*_Gd_1–*x*_(OH)_3_

To further examine whether energy transfer processes between Eu­(III) ions occur in the aforementioned samples, a sample set with the resonance energy transfer (RET) pair Tb­(III)/Eu­(III), in which Tb­(III) would act as the energy donor and Eu­(III) as the energy acceptor, was investigated. The Tb­(III) → Eu­(III) RET is widely studied in literature and used for example to determine interionic distances or binding motifs.^39–41^ In Figure 3, the luminescence spectra of the Tb_*x*_Eu_0.0005*x*_Gd_1–*x*_(OH)_3_ samples are shown. An excitation wavelength of 377 nm was used to excite primarily the Tb­(III) ions. For *x*(Tb) < 0.5, the observed luminescence spectra are governed by the Tb­(III) emission bands of the ^5^D_4_ → ^7^F_3–6_ transitions. At *x* ≥ 0.55 however, additional Eu­(III) luminescence is visible, which overlaps with the ^5^D_4_ → ^7^F_4_ and ^5^D_4_ → ^7^F_3_ emission of Tb­(III) and becomes more pronounced at increasing Tb­(III) content (cf. Figure 3B,D). Additionally, similar to the Eu_*x*_Gd_1–*x*_(OH)_3_ samples, the highest Tb­(III) luminescence intensity was observed around *x*_Tb_ = 0.3. These findings suggest that a similar concentration quenching process as for Eu­(III) in the Eu_*x*_Gd_1–*x*_(OH)_3_ samples also occurs for Tb­(III). For three selected samples with *x*_Tb_ = 0.10, 0.35 and 0.60, additionally a decay curve of the Tb­(III) luminescence was measured. The decay time gradually decreased from (850 ± 20) μs at *x*_Tb_ = 0.10 to (640 ± 10) μs at *x*_Tb_ = 0.35 to (200 ± 20) μs at *x*_Tb_ = 0.60, which further indicates that concentration quenching occurs.

**Figure 3:**
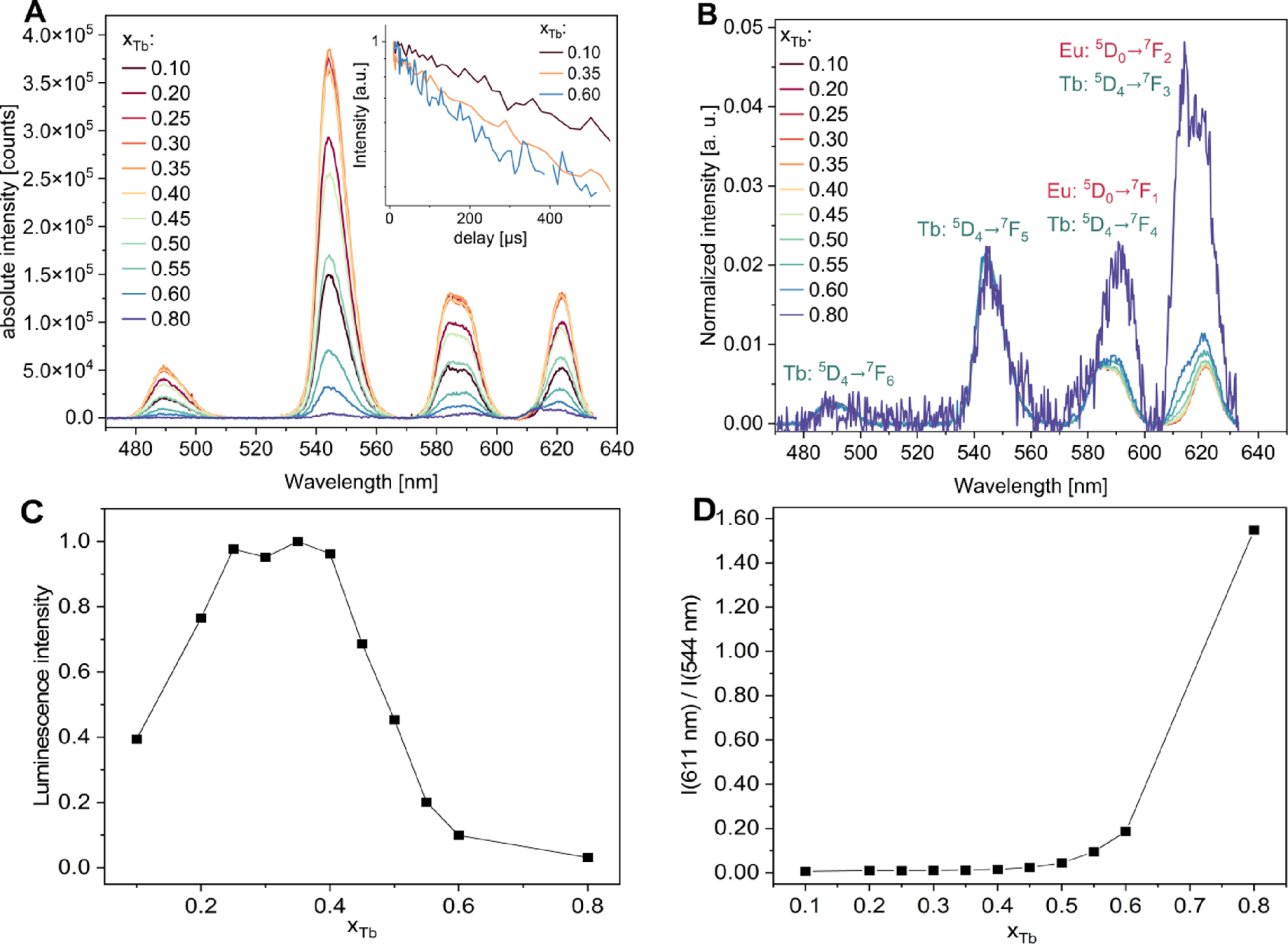
Luminescence spectra of Tb_*x*_Eu_0.0005*x*_Gd_1–*x*_(OH)_3_ at 293 K at a gate delay of 10 μs with different Tb­(III) contents, excited at 377 nm. The spectra are shown with absolute luminescence intensities (A) and normalized to the area of the ^5^D_4_ → ^7^F_5_ transition of Tb­(III) (B). The inset in panel (A) shows the Tb­(III) luminescence decay curves for *x*_Tb_ = 0.10, 0.35, and 0.60. The luminescence intensities at each molar ratio of Tb­(III) (C) and the ratio of the luminescence intensity at 611 nm (representative of the Eu­(III) emission) and the luminescence intensity at 544 nm (representative of the Tb­(III) emission) at different Tb­(III) contents (D) are displayed.

Furthermore, as the Tb­(III)/Eu­(III) ratio is the same in all samples, and as Eu­(III) shows only weak absorption at the used excitation wavelength, the observed Eu­(III) luminescence signal at high values for *x* cannot be explained solely from directly excited Eu­(III) ions. It is tempting to attribute the larger part of the observed Eu­(III) luminescence to a Tb­(III) → Eu­(III) energy transfer. Consequently, energy transfer processes between identical Ln­(III) ions (i.e., between multiple Eu­(III) ions in Eu_*x*_Gd_1–*x*_(OH)_3_ or between multiple Tb­(III) ions and Eu­(III) ions in Tb_*x*_Eu_0.0005*x*_Gd_1–*x*_(OH)_3_) are possible in the studied hydroxide.

### Luminescence Decay Curves of Eu_*x*_Gd_1–*x*_(OH)_3_

In contrast to intensity measurements the luminescence kinetics are less prone to the limitations mentioned for measuring in dispersions (vide supra). In the case of a concentration quenching based on a homo energy transfer (where Eu­(III) ions are both the energy donor and acceptor) the luminescence decay deviates from a first order decay kinetic (monoexponential form) and follows instead the equation derived by Inokuti and Hirayama:^25,42^

$$I\left(t\right)=I\left(0\right)\exp\left\{-\frac{t}{\tau_{0}}-Q{\left(\frac{t}{\tau_{0}}\right)}^{3/S}\right\}+y_{0}$$ 1

Here *I*(*t*) denotes the luminescence intensity at the time *t* after the excitation, *I*(0) denotes the initial luminescence intensity at *t* = 0, *y*_0_ the background signal and τ_0_ the “unquenched“ decay time for an isolated Eu­(III) ion (no quenching by Eu­(III)→Eu­(III) energy transfer). *S* indicates the type of interaction for the Eu­(III) → Eu­(III) energy transfer with *S* = 3 for exchange, *S* = 6 for dipole–dipole interaction, *S* = 8 for dipole–quadrupole interaction and *S* = 10 for quadrupole–quadrupole interaction.^25,42^ *Q* is the quenching parameter and depends on the Eu­(III) concentration *c*(Eu) per Å^3^ and the critical Eu­(III)–Eu­(III) distance *R*_0_, at which the quenching efficiency is 50%:

$$Q=\frac{4}{3}\pi^{1.5}{R_{0}}^{3}c(\mathrm{Eu})$$ 2

The decay curves for all Eu_*x*_Gd_1–*x*_(OH)_3_ samples were evaluated in a global fit according to eq 1 assuming a common value for τ_0_ and *S* for all samples. The decay curves and the resulting fit are displayed in Figure 4. It can be seen, that as *x_Eu_* increases the luminescence decays faster. The fits yielded a value of 6.1 ± 0.1 for *S*, which indicates that in the investigated mixed hydroxides the Eu­(III) → Eu­(III) energy transfer occurs via dipole–dipole interaction.

**Figure 4:**
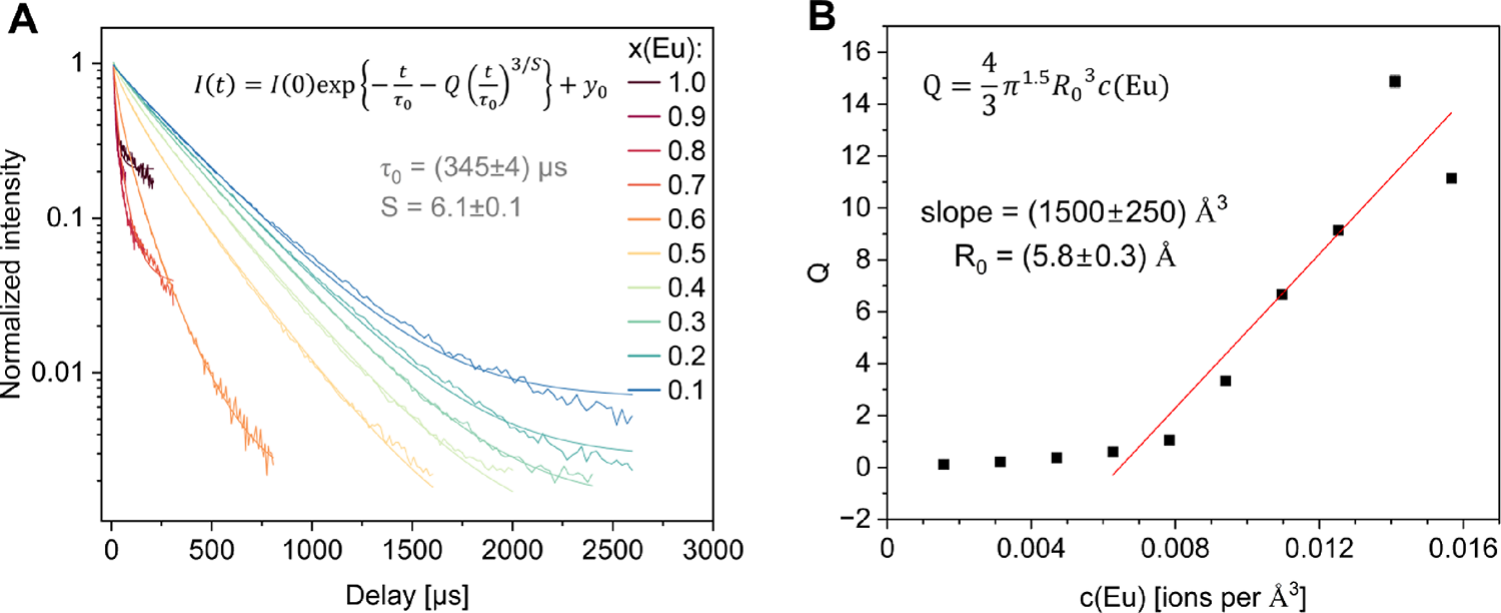
Luminescence decay curves of the Eu_*x*_Gd_1–*x*_(OH)_3_ samples with the respective fit curves according to eq 1 using τ_0_ and *S* as global parameters (A) and linear fit of the obtained quenching parameter *Q* over the average Eu­(III) concentration. (B) Only the samples with *x* ≥ 0.4, in which a significant energy transfer was observed, were included in the fit in panel (B) using eq 4 (or see the inset in panel (B)).

According to eq 2 the critical Eu­(III)–Eu­(III) distance *R*_0_ can be calculated from *Q* if the Eu­(III) concentration per Å^3^ is known. As the analyzed hydroxides are expected to be amorphous, only an estimate of *R*_0_ can be obtained. Here, it was assumed that the ions in the present samples are arranged in a similar manner as in the crystal structure of crystalline Eu­(OH)_3_ given by Mullica et al. with 2 Ln­(III) ions per unit cell and a unit cell volume of 127.644 Å^3^.^43^ Since the crystal structure of crystalline Gd­(OH)_3_ is very similar to that of Eu­(OH)_3_ (with a unit cell volume of 125.958 Å^3^)^44^ and the normalized Eu­(III) luminescence spectra of all samples are essentially identical (cf. Figure 2B), no significant changes in the structure with varying Eu­(III) molar ratios are expected. Taking into account the above-mentioned values, the Eu­(III) concentration per Å^3^ can be estimated according to eq 3:

$$c\left(\mathrm{Eu}\right)\approx \frac{2x}{127.644{Å}^{3}}$$ 3

The critical quenching distance *R*_0_ can then be determined from the slope of a plot of *Q* over *c*(Eu), which is shown in Figure 4B:

$$\mathrm{slope}=\frac{4}{3}\pi^{1.5}{R_{0}}^{3}$$ 4

As shown in Figure 4B, *Q* is close to 0 for the samples with the lowest Eu­(III) content, indicating that the average Eu­(III)–Eu­(III) distance in those samples is too large for an effective energy transfer to occur. Hence, only the samples in which a significant energy transfer was observed (*x* ≥ 0.4) were included in the fit. From this fit, *R*_0_ was determined to be 5.8 ± 0.3 Å which is well within the distance range reported in the literature for a dipole–dipole interaction of Eu­(III).^32,36,38,45^ It must be noted however, that this value is only an estimate based on the crystal structure of crystalline Eu­(OH)_3_. For the amorphous samples this can only be seen as one limiting case.

### Temperature Dependence

To further investigate the energy transfer mechanism the temperature dependence of the luminescence kinetics was investigated. Because of experimental constrains for those experiments a higher total Eu­(III) concentration was required. Therefore, an additional sample with *x* = 1 (pure Eu­(OH)_3_) with a Eu­(III) starting concentration of 1 × 10^–3^ M was prepared and measured at different temperatures between 4 K ≤ *T* ≤ 293 K. The decay curves were fitted with eq 1 as described above. Because reference experiments^46^ suggested that the decay time of the isolated Eu­(III) ion τ_0_ is largely independent of temperature, the fit was done with a global τ_0_ for all temperatures and using *S* = 6. The fit yielded a decay time τ_0_ of 325 ± 4 μs, which is in line with the results for the set of Eu_*x*_Gd_1–*x*_(OH)_3_ described above. The variation of the quenching parameter *Q* with temperature is shown in Figure 5D. A sharp increase of *Q* between 210 and 250 K was observed, indicating that at T≤ 210 K the energy transfer between Eu­(III) ions becomes significantly less effective.

**Figure 5:**
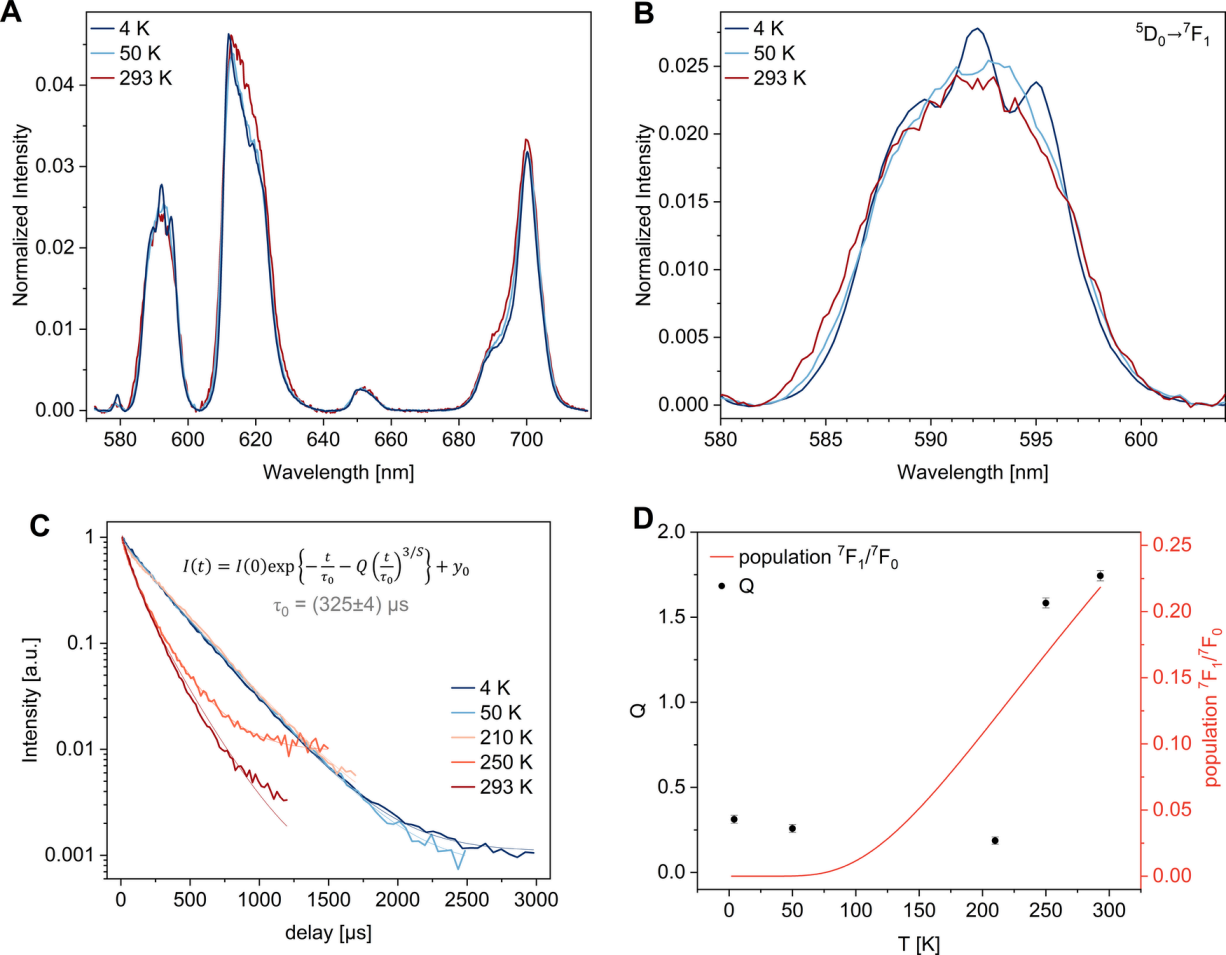
Top: Luminescence spectra of Eu­(OH)_3_ at a gate delay of 10 μs at different temperatures normalized to the area of the ^5^D_0_ → ^7^F_1_ transition. Full emission spectra (A) and only the ^5^D_0_ → ^7^F_1_ emission (B) are shown. The spectra at 4 K were measured with a slit width of 25 μm and 1000 accumulations, while the spectra at 50 and 293 K were measured with a slit width of 400 μm and 50 accumulations. Bottom: Luminescence decay curves with the respective fit curves according to eq 1 using *S* = 6 and τ_0_ as a global parameter (C) and the obtained quenching parameter *Q* for Eu­(OH)_3_ with a starting concentration of 1 × 10^–3^ M Eu­(III) at different temperatures (black circles) and ratio of Eu­(III) ions in the ^7^F_1_ and ^7^F_0_ level at the respective temperature (red curve). Between 210 and 250 K, a sharp increase of *Q* (and hence of the energy transfer rate) can be observed. This corresponds to a ^7^F_1_/^7^F_0_ ratio of 0.17 at 250 K (i.e., every ∼6th ion is in the ^7^F_1_ level) (D).

For Eu­(III) the transition between the ground state (^7^F_0_) and the excited ^5^D_0_ level of Eu­(III) is strictly forbidden which is translating into a very small transition probability and subsequently to a low efficiency for a resonance energy transfer. In contrast, the magnetic ^5^D_0_ ⇆ ^7^F_1_ transitions and the ^5^D_0_ ⇆ ^7^F_2_ transitions have a higher transition probability (in absorption and emission, respectively) and thus it is tempting to assume that Eu­(III) ions in thermally populated ^7^F_1_ Stark levels (and potentially also of the ^7^F_2_) are involved in the Eu­(III) → Eu­(III) energy transfer via dipole–dipole interaction. The ratio between the population in the ^7^F_1_ and ^7^F_0_ is ∼ $\exp\left(-\frac{\Delta E}{kT}\right)$ , with Δ*E* = 310 cm^–1^ as the energy difference between the ^7^F_0_ and lowest-lying ^7^F_1_ Stark level (^7^F_1,1_) for the investigated Eu­(OH)_3_ sample (Δ*E* was determined from the luminescence spectrum shown in Figure 5A at 4 K, which allows the determination of the energy difference between the ^7^F_0_ and ^7^F_1,1_ level). As depicted in Figure 5D, the ^7^F_1,1_/^7^F_0_ ratio of thermal population at 250 K is 0.17, which appears to be the necessary value for significant concentration quenching to occur in the investigated Eu­(OH)_3_ sample. Additionally, at higher temperatures the energy transfer may be improved by overcoming small energy mismatches between Eu­(III) ions with slightly different chemical environment via the assistance of lattice vibrations.

It is noticeable, that the quenching parameter *Q* at 293 K for the sample investigated in this section, which was prepared with an initial Eu­(III) concentration of 1 × 10^–3^ M (*Q* ∼ 1.7) deviates from the value of the Eu­(OH)_3_ sample from the first sample set at 293 K, which was prepared with an initial Eu­(III) concentration of 5 × 10^–5^ M (*Q* ∼ 11). This suggests that the different experimental conditions, such as the Eu­(III) concentration, pressure and aging time, gave rise to structural differences between the formed hydroxides, e.g. in the particle size, degree of crystallinity or the Eu­(III)–Eu­(III) distances. However, the high Eu­(III) concentration of 1 × 10^–3^ M as well as the applied vacuum were necessary for the temperature-dependent measurement setup. Nonetheless, we expect that the general trend with temperature and the involvement of thermally populated ^7^F_1_ and ^7^F_2_ levels in the quenching process are comparable in the samples prepared with 5 × 10^–5^ M Ln­(III) and atmospheric pressure.

### Quenching Mechanism

The concentration quenching in the present study can primarily be attributed to energy migration. Here, the excitation energy is transferred from one Eu­(III) ion to the next, until it eventually reaches a quenching center. Such quenching centers could be impurity ions or Eu­(III) ions in a slightly different coordination environment, such as on defect sites or edge positions. In the literature, both Eu­(III) → Eu­(III) energy transfer via (super)­exchange^30,31,34,35^ and via multipolar interaction^32,33,36^ are described, and the nature of the transfer interaction appears to be dependent on the material. For the investigated Eu_*x*_Gd_1–*x*_(OH)_3_ samples, the fit of the luminescence decay curves according to eq 1 yielded *S* = 6.1 ± 0.1, suggesting that dipole–dipole interaction is the dominant transfer mechanism in these samples. Moreover, the temperature-dependent measurements indicate a significant involvement of Eu­(III) ions in thermally populated ^7^F_1_ and ^7^F_2_ levels in the quenching process. The estimated critical Eu­(III)–Eu­(III)-distance *R*_0_ of 5.8 ± 0.3 Å implies, that the energy is transferred mainly to nearest-neighbor Eu­(III) ions. Cross-relaxation processes may also contribute to the observed luminescence quenching. However, the probability of such processes is expected to be small for Eu­(III), as they require either two neighboring Eu­(III) ions in the ^5^D_*J*_ manifold^26,28,29^ or the simultaneous involvement of three Eu­(III) ions with a remaining energy mismatch of ∼2000 cm^–1^.^24,25^

Additionally, it should be noted, that the unquenched decay time τ_0_ at infinitely low Eu­(III) content (345 ± 4 μs based on the fit shown in Figure 4A) is still distinctly shorter than one would expect for Eu­(III) ions that are free from external deactivation. In such a case a decay time in the millisecond range would have been found. Judd–Ofelt analysis^47^ using the luminescence spectra of the Eu_*x*_Gd_1–*x*_(OH)_3_ samples yielded a theoretical unquenched luminescence decay time of 4.9 ms, from which a luminescence quantum yield of Φ_lum_ = τ_measured_/τ_theo_ = 7% can be derived for *x* → 0, when no energy transfer between Eu­(III) ions is possible. From the short decay time τ_0_ and low quantum yield Φ_lum_ it can be concluded that in addition to the concentration quenching also other deactivation processes that are independent of the Eu­(III) concentration occur. It is attractive to attribute this additional luminescence quenching to an energy transfer to O–H oscillators such as water molecules and OH^–^ ions.^22,23^

## Conclusions

The luminescence quenching mechanism in amorphous Eu_*x*_Gd_1–*x*_(OH)_3_ was investigated. TRLFS experiments at different molar fractions of Eu­(III) (0.1 ≤ *x* ≤ 1.0) showed, that the short luminescence decay time and low luminescence intensity of the investigated hydroxides are primarily caused by energy transfer processes between neighboring Eu­(III) ions. The use of the RET pair Tb­(III)/Eu­(III) enabled us to detect the increase in Eu­(III) luminescence with increasing Tb­(III) and Eu­(III) content and hence offered complementary information, that energy transfer between lanthanide ions occurs in the investigated materials. In detail, for Eu_*x*_Gd_1–*x*_(OH)_3_ excitation energy could dissipate via energy migration through the Eu­(III) sublattice until a quenching center is reached, or to a lesser extent via cross-relaxation involving one excited Eu­(III) ion and two Eu­(III) ions in the ground state or via cross-relaxation between two excited Eu­(III) ions. The fit of the luminescence decay curves implies that the energy transfer processes occur via dipole–dipole interaction with an estimated critical Eu­(III)–Eu­(III)-distance *R*_0_ of 5.8 ± 0.3 Å. At *x* = 1, the energy transfer rate significantly decreases below 250 K, at which roughly every sixth Eu­(III) ion is in a thermally populated ^7^F_1_ level, which emphasizes the relevance of Eu­(III) ions in the ^7^F_1_ and ^7^F_2_ levels for the resonance transfer. Additionally, concentration-independent quenching processes, most likely due to an energy transfer to the overtone vibrations of the OH^–^ ions and potentially surrounding H_2_O molecules occur.^22,23^

To summarize, the strong luminescence quenching in Eu­(OH)_3,am_ can be explained by a combination of energy transfer to O–H vibrations and an additional, very efficient energy transfer between Eu­(III) ions. These findings may also offer relevant insights for the luminescence and energy transfer processes in other Eu-rich precipitates that form in aqueous media, such as mixed Eu–Ca–Si precipitates that can form under certain conditions in cement pore water.^48,49^

## References

[1] Bünzli J.-C. G. (2016). Lanthanide
light for biology and medical diagnosis. J.
Lumin..

[2] Bünzli J. C. G., Piguet C. (2005). Taking advantage of luminescent lanthanide ions. Chem. Soc. Rev..

[3] Reisfeld R. (2015). Optical properties
of lanthanides in condensed phase, theory and applications. AIMS Press..

[4] Binnemans K. (2015). Interpretation
of Europium­(III) spectra. Coord. Chem. Rev..

[5] Hagan A. K., Zuchner T. (2011). Lanthanide-based time-resolved
luminescence immunoassays. Anal. Bioanal. Chem..

[6] Allen K. N., Imperiali B. (2010). Lanthanide-tagged proteins—an illuminating partnership. Curr. Opin. Chem. Biol..

[7] Tits, J. , Wieland, E. Actinide sorption by cementitious materials. PSI Report 18–02; Paul Scherrer Institut: Villigen, Switzerland, 2018.

[8] Kim H.-K., Cho H.-R., Cha W. (2024). Solubility
of Trivalent Am, Eu, and
Sm in the Synthetic KAERI Underground Research Tunnel Groundwater. Journal of Nuclear Fuel Cycle and Waste Technology(JNFCWT).

[9] Murota K., Aoyagi N., Mei H., Saito T. (2023). Hydration states of
europium­(III) adsorbed on silicas with nano-sized pores. Appl. Geochem..

[10] Lothenbach B., Nonat A. (2015). Calcium silicate hydrates:
Solid and liquid phase composition. Cem. Concr.
Res..

[11] Giffaut E. (2014). Andra thermodynamic database for performance assessment: ThermoChimie. Appl. Geochem..

[12] Jordan N., Thoenen T., Spahiu K., Kelling J., Starke S., Brendler V. (2024). A critical review of
the solution chemistry, solubility,
and thermodynamics of europium: Recent advances on the Eu­(III) hydrolysis. Coordination Chemistry Reviews.

[13] Parkhurst, L. , Appelo, C. Description of input and examples for PHREEQC version 3: a computer program for speciation, batch-reaction, one-dimensional transport, and inverse geochemical calculations. Techniques and Methods, book 6, chap. A43; US Geological Survey: Denver, USA., 2013. [Online]. Available: https://pubs.usgs.gov/tm/06/a43/.

[14] Andolina C. M., Mathews R. A., Morrow J. R. (2009). Solution chemistry of Europium­(III)
aqua ion at Mmcromolar concentrations as probed by direct excitation
luminescence spectroscopy. Helv. Chim. Acta.

[15] Kim H. K., Choi S., Jung E. C., Cho H. R., Yun J. Il, Cha W. (2018). TRLFS study
of hydrolyzed Eu­(III) species. J. Lumin..

[16] Runde W., Van Pelt C., Allen P. O. (2000). Spectroscopic
characterization of
trivalent f-element (Eu, Am) solid carbonates. J. Alloys Compd..

[17] Takahashi Y., Kimura T., Kato Y., Minai Y., Tominaga T. (1998). Characterization
of Eu­(III) species sorbed on Silica and Montmorillonite by laser-induced
fluorescence spectroscopy. Radiochim. Acta.

[18] Plancque G., Moulin V., Toulhoat P., Moulin C. (2003). Europium speciation
by time-resolved laser-induced fluorescence. Anal. Chim. Acta.

[19] Ishida K. (2012). Surface speciation of Eu3+ adsorbed on kaolinite by
time-resolved
laser fluorescence spectroscopy (TRLFS) and parallel factor analysis
(PARAFAC). J. Colloid Interface Sci..

[20] Zenker S. (2025). Complexation of Ln­(III) ions by gluconate: joint investigation applying
TRLFS, CE-ICP-MS, NMR, and DF calculations. Inorg. Chem..

[21] Supkowski R. M., Horrocks W. D. W. (2002). On the determination of the number
of water molecules,
q, coordinated to Europium­(III) ions in solution from luminescence
decay lifetimes. Inorg. Chim. Acta.

[22] Marmodée B., Jahn K., Ariese F., Gooijer C., Kumke M. U. (2010). Direct
spectroscopic evidence of 8- and 9-fold coordinated Europium­(III)
species in H_2_O and D_2_O. J. Phys. Chem. A.

[23] Kimura T., Choppin G. R. (1994). Luminescence study
on determination of the hydration
number of Cm­(III). J. Alloys Compd..

[24] Van
Uitert L. G., Johnson L. F. (1966). Energy transfer between rare-earth
ions. J. Chem. Phys..

[25] Tan P. M. (2020). New insights on the
energy transfer mechanisms of Eu-doped CdS quantum
dots. Phys. Chem. Chem. Phys..

[26] Manigault P., Summers C. J., Stoffers C. (2002). Second-order luminescent saturation
effects in SrGa_2_S_4_:Eu. Appl. Phys. Lett..

[27] Yusenko K. V. (2021). Local structure of Europium-doped
luminescent Strontium Fluoride
nanoparticles: comparative X-ray absorption spectroscopy and diffraction
study. ChemNanoMat.

[28] Hansen P. A., Granerød C. S., Prytz Ø., Nilsen O. (2019). Controlling luminescence
and quenching mechanisms in subnanometer multilayer structure of Europium
Titanium Oxide thin films. J. Lumin..

[29] Tallant D. R., Seager C. H., Simpson R. L. (2002). Energy
transfer and relaxation in
europium-activated Y2O3 after excitation by ultraviolet photons. J. Appl. Phys..

[30] Buijs M., Blasse G. (1986). Luminescence and energy
migration in a one-dimensional
system: EuMgB5O10. J. Lumin..

[31] Kononets N. V. (2017). Processes of energy
migration in mixed Europium–Lanthanum
Magnesium Borate nanocrystals. Spectrosc. Lett..

[32] Han B., Zhang J., Wang Z., Liu Y., Shi H. (2014). Investigation
on the concentration quenching and energy transfer of red-light-emitting
phosphor Y2MoO6:Eu3+. J. Lumin..

[33] Zhang Y., Xu J., Yang B., Cui Q., Tian T. (2018). Luminescence properties
and energy migration mechanism of Eu^3+^ activated Bi_4_Si_3_O_12_ as a potential phosphor for white
LEDs. Mater. Res. Express.

[34] Zhang W. (1998). Preparation and size
effect on concentration quenching of nanocrystalline
Y2SiO5:Eu. Chem. Phys. Lett..

[35] Van
Uitert L. G., Dearborn E. F., Rubin J. J. (1967). Mechanisms of energy
transfer involving trivalent Eu and Nd. J. Chem.
Phys..

[36] Abhilash
Kumar R. G., Hata S., Ikeda K., Gopchandran K. G. (2015). Luminescence
dynamics and concentration quenching in Gd2–xEuxO3 nanophosphor. Ceram. Int..

[37] Hayakawa T., Nogami M. (2001). Energy migration of
the local excitation at the Eu3+
site in a Eu–O chemical cluster in sol-gel derived SiO2:Eu3+
glasses. J. Appl. Phys..

[38] Kononets N. V., Seminko V. V., Maksimchuk P. O., Bespalova I. I., Klochkov V. K., Malyukin Yu. V. (2018). Energy migration
processes in phosphate
nanocrystals: Size and dimensionality dependence. Low Temperature Physics.

[39] Nonat A., Liu T., Jeannin O., Camerel F., Charbonnière L.
J. (2018). Energy
transfer in supramolecular heteronuclear lanthanide dimers and application
to Fluoride sensing in water. Chem. - Eur. J..

[40] Brittain H. G. (1990). Intermolecular
energy transfer between lanthanide complexes. 10. Tb­(III) donor and
Eu­(III) acceptor complexes of triethylenetetra aminehexaacetic acid. J. Coord. Chem..

[41] Kumke M. U., Eidner S., Krüger T. (2005). Fluorescence
quenching and luminescence
sensitization in complexes of Tb3+ and Eu3+ with humic substances. Environ. Sci. Technol..

[42] Inokuti M., Hirayama F. (1965). Influence of energy
transfer by the exchange mechanism
on donor luminescence. J. Chem. Phys..

[43] Mullica D.
F., Milligan W. O., Beall G. W. (1979). Crystal structures of Pr­(OH)­3, Eu­(OH)­3
and Tm­(OH)­3. Journal of Inorganic and Nuclear
Chemistry.

[44] Beall G. W., Milligan W. O., Wolcott H. A. (1977). structural trend
in the lanthanide
trihydroxides. J. Inorg. Nucl. Chem..

[45] Berdowski P. A. M., Buijs M., Blasse G. (1985). Energy migration
in Eu3+ compounds;
its dependence on dimensionality and Eu3+-Eu3+-distance. J. Phys. Colloq..

[46] Kuke S., Marmodée B., Eidner S., Schilde U., Kumke M. (2010). Intramolecular
deactivation processes in complexes of salicylic acid or glycolic
acid with Eu­(III). Spectrochim. Acta A Mol.
Biomol. Spectrosc..

[47] Ćirić A., Stojadinović S., Sekulić M., Dramićanin M. D. (2019). JOES: An
application software for Judd-Ofelt analysis from Eu3+ emission spectra. J. Lumin..

[48] Dettmann S., Huittinen N. M., Jahn N., Kretzschmar J., Kumke M. U., Kutyma T., Lohmann J., Reich T., Schmeide K., Shams Aldin Azzam S., Spittler L., Stietz J. (2023). Influence of gluconate on the retention of Eu­(III), Am­(III), Th­(IV),
Pu­(IV), and U­(VI) by C-S-H (C/S = 0.8). Front.
Nucl. Eng..

[49] Schlegel M. L., Pointeau I., Coreau N., Reiller P. E. (2004). Mechanism of Europium
retention by Calcium Silicate Hydrates: An EXAFS study. Environ. Sci. Technol..

